# Root Resorption of the Permanent Central Incisor through Ectopic Eruption of the Maxillary Canine

**DOI:** 10.1155/2023/2602899

**Published:** 2023-06-21

**Authors:** Maria Pires Machado Paixão, Kelly Maria Silva Moreira, Ricardo Scarparo Navarro, Suzana Cavalcanti Monteiro de Oliveira, José Carlos Pettorossi Imparato, Juliana Braga Reis

**Affiliations:** Graduate Program in Pediatric Dentistry, São Leopoldo Mandic Faculty, Institute and Research Center São Leopoldo Mandic, Campinas, São Paulo, Brazil

## Abstract

Although ectopic eruption of the maxillary canine has a low prevalence, a late diagnosis can have serious consequences. A careful clinical examination, assisted with radiographic examination, ensures early diagnosis, facilitates planning, and minimizes possible adverse consequences. This study reports a case of ectopic eruption of the permanent maxillary canine, with complete root resorption of the central permanent incisor, the consequences of which caused functional, aesthetic, and psychological harms to the patient. The procedures used included canine ectopic remodeling of the ectopic canine in the central incisor and orthodontic correction, which treated the anomaly and rebuilt the patient's self-esteem.

## 1. Introduction

Human dentition is characterized by precise and complex biological processes involving the substitution of deciduous teeth with permanent teeth. However, as in all physiological processes, dentition development can cause some imperfections, and during mixed dentition, odontogenic changes can occur, which may appear with distinct degrees of severity [1].

The impaction of the canines is one such change. These canines are classified as teeth that do not erupt after the total formation of their roots, or those that, six months after the eruption of the homologous tooth, with its root completely formed, did not erupt [2]. The etiology can be explained by genetic or hereditary factors, but is strongly influenced by environmental factors, such as soft tissue lesions, hard tissue bodies, or developmental pathologic entities [2–4]. Buccal impacted canines are usually associated with maxillary transverse discrepancies, and palatally impacted canines are associated with a pattern of dental anomalies, such as maxillary lateral incisor agenesis and mandibular premolar distoangulation [5, 6].

The prevalence of canine impaction ranges from 0.92% to 5.2%, with the upper teeth being the most affected [7]. Despite the low prevalence of this alteration, the impaction of canines can have severe consequences, such as the absence of an impacted tooth in the dental arch and root resorption of the permanent adjacent teeth, which may in turn leads to functional, aesthetic, and psychological changes [8–10].

The prevalence of root resorption in central incisors range from 1.19% to 35.06% and it is correlated with their contact with the maxillary canine during eruption. It is considered severe in 30.9% of the cases [11]. Different prevalence values of impacted canines and central root resorptions were found ranging from 4.0% to 46.67%, and severe root resorption was demonstrated in 2.27–50.00% of cases across the studies that used only cone-beam computed tomography. It can be explained because cone-beam computed tomography provides more accurate imaging than conventional two-dimensional radiographs. The superimposition of the incisor and the canine as well as the magnification error and distortions due to root angulation, commonly observed among two-dimensional imaging techniques [12].

Leaving impacted canines without proper intervention results in serious damage of the adjacent teeth, and the predisposing factors associated with root resorption are the permanent canine rotation, separated root configuration of the first premolar, contact relationship, and area of contact with adjacent teeth [13]. The study of these changes is of utmost importance in dentistry, given that the success of the treatment and the prognosis depend on early diagnosis for intervention, when necessary, with the best possible therapeutic conduct, which are the rapid maxillary expansion and deciduous canine extraction, to foster the development of occlusion within the parameters of normality [8, 10, 14]. For a precise diagnosis, the clinical examination should be complemented by a radiographic evaluation, since there are concerns about the possible consequences related to the ectopic eruption of the impacted canines, which can only be seen through radiographic exams [8, 10, 15, 16].

Early diagnosis of palatally impacted canine and evaluation of the risk factors for root resorption together with proper interceptive treatment are crucial to prevent future complications, such as severe root resorption of the permanent teeth, that might compromise its viability and longevity. In this context, this study deals with the dental and psychological implications of ectopic eruption of an impacted canine. This study aimed to present a case report of ectopic eruption of a right permanent maxillary canine in an 11-year-old patient, which, due to a late diagnosis, resulted in root resorption and exfoliation of the permanent central incisor. Through this case report, this study seeks to reiterate the importance of the early diagnosis of dental anomalies, which will in turn make it possible to perform opportune interception and prevent complications in the development of dentition.

## 2. Case Presentation

This study was approved by the Research Ethics Committee of São Leopoldo Mandic College, logged under protocol number 0631/2015.

**Figure 1:**
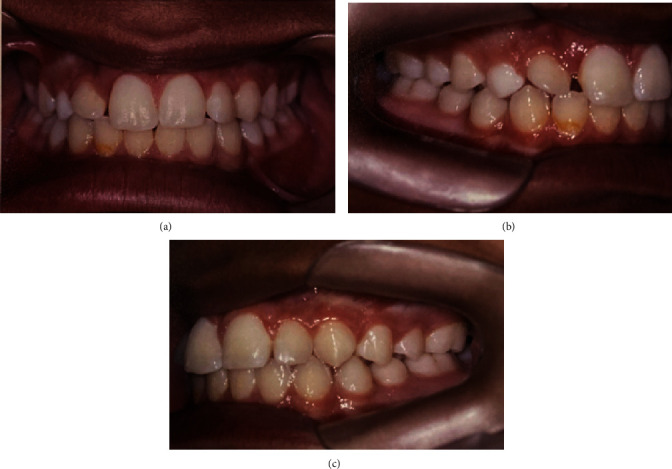
Intraoral photos of the initial clinical condition. (a) Front side. (b) Right side. (c) Left side.

**Figure 2:**
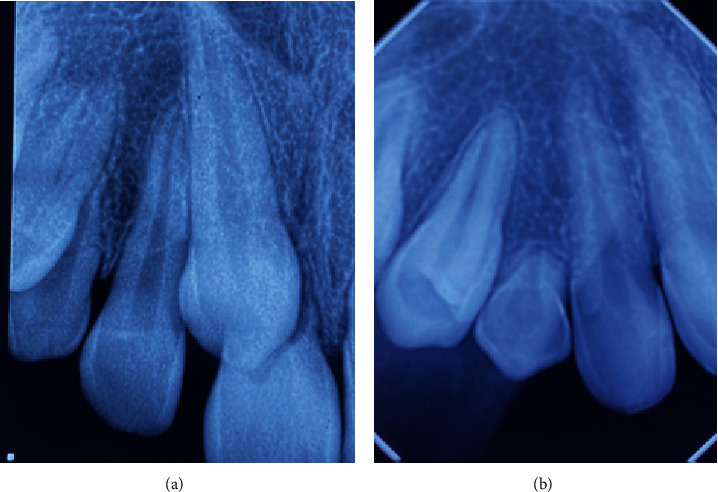
Periapical radiographs. (a): Permanent canine (tooth #13) in ectopic position in the central incisor region (tooth #11). (b) Deciduous canine (element 53) with 1/3 of the root present, and the absence of an image of the permanent canine.

**Figure 3:**
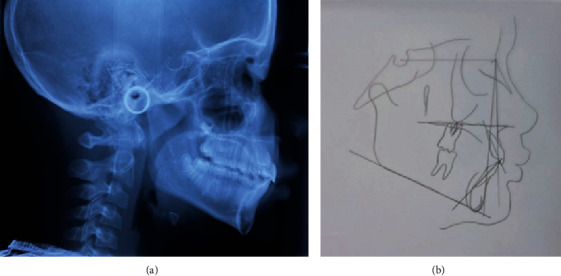
(a) Teleradiograph. (b) Cephalometric tracing.

In May 2012, an 11-year-old patient, L. de F.C., female, together with her guardian, sought dental care, complaining mainly of lateral incisor distoangulation and pathological mobility of the right upper central incisor (tooth #11), and as well as the absence of tooth #13. Visually, there was no clear evidence of any changes (Figure 1). Nevertheless, after clinical examination, the diagnostic hypothesis was that of an ectopic position of the permanent canine, which was suggested due to the difference in palpation between the right and left sides of the canine region. Radiographic examinations and teleradiograph were requested (Figures 2 and 3), which confirmed the diagnostic hypothesis. The patient presented balanced growth, without skeletal discrepancy between the bone bases (SNB 87, SNA 92, and ANB 5) with adequate angulation of the upper and lower incisors (IMPA: 105° and I PP: 122°).

**Figure 4:**
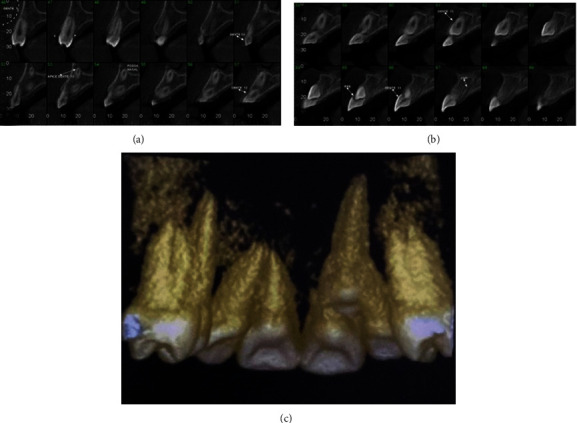
Images of the sagittal cut from the tomography. (a) Tooth #53 still with 1/3 of the root. (b) Root resorption of tooth #11. (c) 3D view.

After confirming the transposition of the canine and the substantial mobility identified in tooth #11, tomography was performed to evaluate the root resorption of tooth #11. Examination showed that the permanent canine was in an ectopic position and had completely resorbed the root of the permanent central incisor (Figure 4). The treatment option proposed and accepted by the patient, and her guardian was orthodontic treatment for realignment, leveling, and re-anatomization of the canine in the central incisor.

**Figure 5:**
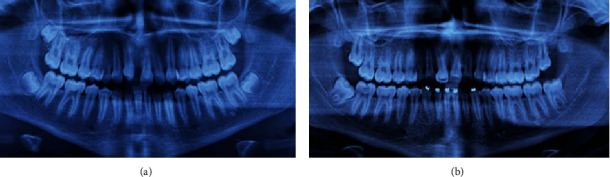
Panoramic radiographs. (a) After orthodontic and aesthetic treatments. (b) Before orthodontic and aesthetic treatments.

After exfoliation of the permanent central incisor (tooth #11) in August 2012, new radiographic examinations were requested to follow-up on the eruption of the ectopic canine (Figure 5(a)). The patient continued to undergo constant follow-up and orthodontic treatment; however, this description is not the core focus of this case report.

**Figure 6:**
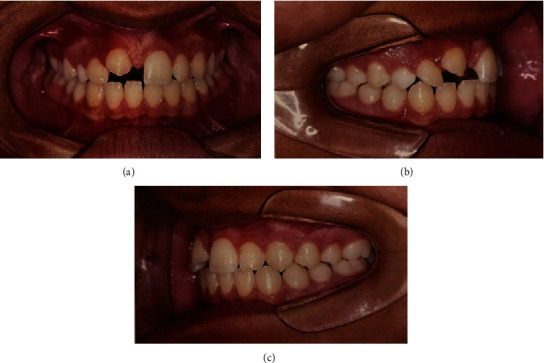
Intraoral photos of the clinical condition. (a) Tooth #13 ectopically erupted. (b) Right side. (c) Left side.

One year later, in August 2013, the canine erupted (Figure 6).

It is important to highlights the extent to which this clinical condition interferes negatively with the self-esteem of the patient. This psychological impact can be clearly observed in the differences in the patient's smile line.

**Figure 7:**
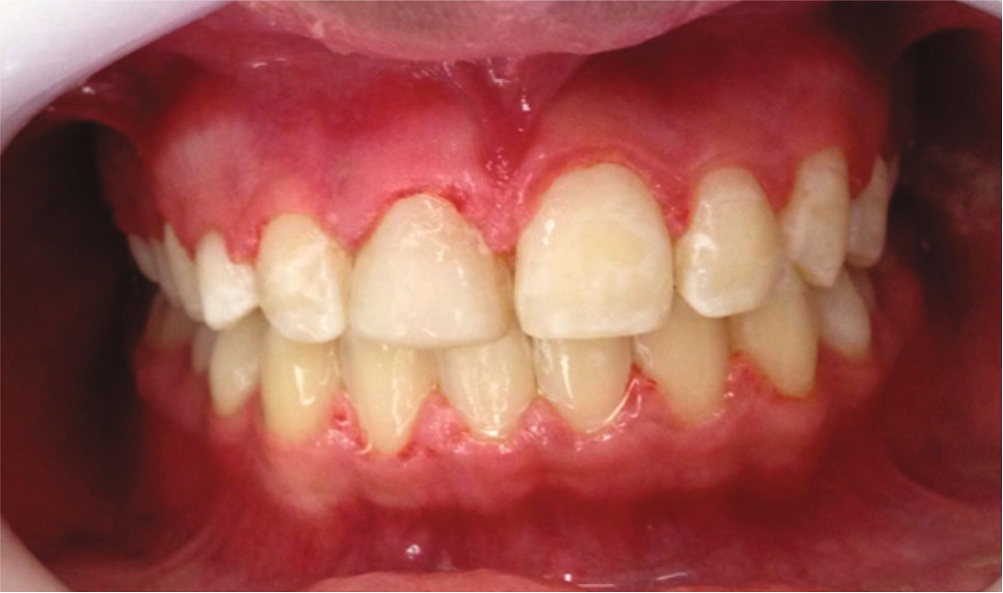
Intraoral photo of aesthetic reshaping with the transformation of the ectopic canine into a permanent central incisor.

In May 2015, near the end of her orthodontic treatment, the patient underwent aesthetic tooth reshaping for re-anatomization of the ectopic canine in the central incisor, performed at the Postgraduate Clinic of the São Leopoldo Mandic College in Belo Horizonte, Brazil, within the course of Dental Pediatrics. The procedure was conducted using diamond-tipped burs. The enamel was conditioned with phosphoric acid at 37% for 20 seconds, the “Ambar” adhesive system from the manufacturer FGM, “Opallis” nanohybrid compound resin from the manufacturer FGM in the colors of EA1, D-Bleach, DA2, and as well as finishing and occlusal adjustment with “Shofu” at a high rotation and carbon (Figures 5(b) and 7).

After completing the orthodontic treatment and bone growth process, the other stages of the treatment plan will be carried out: extraction of tooth #53, a fixed prosthesis upon the osseointegrated implant, and periodontal treatment with gingivectomy for aesthetic reshaping.

## 3. Discussion

In dental pediatrics, we commonly encounter patients in the growth and development process, that is, in a phase of transition and constant change. Thus, dental planning may be limited by some variables, one of which is the patient's current stage of growth. Therefore, it is necessary for dental pediatrics to focus on prevention and early diagnosis to maintain the integrity of the patient's health, and as well as the proper development and functioning of the stomatognathic system.

After the third molars, the permanent canine is the most impacted tooth [3]. In addition, the most common sequel of maxillary ectopic canines is root resorption [17]. Ectopic eruption and the resulting transposition are among the most difficult challenges for orthodontists [18]. In a computerized tomography study, Ericson and Kurol [19] found that 50% of canines were palatally displaced, and the remainder were either buccal or in the line of the arch. Root resorption was found in 38% of lateral incisors and in 9% of central incisors. Ali et al. [20] also observed a higher frequency of displacement to the palate, apically positioned, and in females. When present, resorption mainly affected the lateral incisors with a predominantly mild degree. The absence of prompt treatment can cause resorption in adjacent teeth, but without gender predilection. Nagani et al. observed a higher frequency of unilateral, buccally displaced canines, and found no association between ectopic upper canines and resorption of adjacent teeth. In our case, the patient was female, the ectopic canine was superior, was positioned buccally and apically, and there was marked resorption of the central incisor, evolving with its loss.

Palatally displaced canines are commonly associated with dental anomalies, such as microdontia, conoid lateral incisors, enamel hypoplasia, infraocclusion of deciduous molars, delay in tooth development and eruption, transposition, and tooth agenesis [17]. Odontomas are significantly associated with impacted teeth, mainly affecting the lower canines. Early diagnosis and timely treatment are essential to avoid complications, such as prolonged retention of deciduous teeth and delayed eruption of permanent teeth [21].

Increased mesial angulation and overlapping of the crown of the canine with the root of the lateral incisor are parameters that define the diagnosis of a canine at increased risk of impaction and resorption of the central or lateral incisors. The lateral incisor acts as an eruption guide for the canines. As soon as the canine germ touches the lateral root, it verticalizes. If there is a genetic alteration or lack of space, it does not verticalize and is angled mesially, overlapping the lateral and/or central root on the buccal (more common when there is a lack of space) or palatine side (due to genetic predisposition, associated with lateral incisor agenesis, premolar distoangulation) [2, 22, 23].

Careful monitoring of the presence and position of unerupted canines in developing dentition is essential. The canine moved from a palatal to buccal position during the last stages of its eruption. Late diagnosis can prolong treatment time and increase the risk of root resorption in adjacent teeth [24]. Early extraction of the deciduous canine, mesial position of the crown, and mesial inclination of the displaced canine are the most important variables that predict the possible spontaneous eruption of the impacted tooth [25]. Initial canine angulation and space assessments may be used as predictors of successful palatally displaced canines' eruption. Initial canine angulation and spatial assessments can be used as predictors of successful eruption of palatally displaced canines, with the angle formed between the maxillary canine and the condylar line being the most important (Angle A). No difference was found between early extraction of the primary canine only and joint extraction with the canine and primary first molar as an impaction treatment approach [26].

Cannavale et al. described the early diagnosis and treatment of an ectopic premolar, preventing a complete transposition and repositioning the involved teeth to their normal anatomical positions in the dental arch. Transposition correction is important and should be done as soon as possible. The pediatric dentist must correctly identify the transpositions and understand the therapeutic possibilities.

As observed in this case report, the severe consequences of the ectopic eruption of the permanent canine confirm the importance of periodic follow-up of the patient with rigorous clinical examinations and, if necessary, complementary examinations, such as radiographic examinations, which make it possible to reach an early diagnosis, as well as safe and efficient treatment planning, with less damage and interventions [8, 27]. In addition, this will allow the prediction of future problems of occlusion and the development of permanent teeth [28, 29]. Interceptive treatment involves rapid expansion of the maxilla when associated with atresia of the maxilla with or without extraction of the deciduous canine. Traction biomechanics can also be used successfully for repositioning impacted and transposed teeth [30].

Another equally important alteration, although rare, to be diagnosed early is permanent canine agenesis [31]. It can often be confused with ectopic eruption because both lead to prolonged impaction of the primary canine.

Radiographs are valuable complementary examinations in oral health care, but their indications should be individualized for each patient and not based on age. Panoramic radiography is indicated in new patients in the mixed dentition phase after eruption of the first permanent molars. However, the professional must judge the need and type of radiographic examination necessary for the assessment and/or monitoring of the child's growth and development [32].

Panoramic radiography is the first choice of complementary exam for the early diagnosis of permanent canine impaction and follow-up of the case, with cone-beam computed tomography being preferred for localization of the impacted tooth, detailed visualization of the relationship with adjacent anatomical structures, and prevention of complications [7].

Many authors agree on the importance of early diagnosis, and minimal intervention is widely defended in dentistry. However, the idea of creating a protocol for radiographic examinations in dental pediatrics remains highly controversial [8, 10, 27]. Nevertheless, the patient in this case report presented no clinical changes before the incisor presented with advanced root resorption, which could have been avoided if the diagnosis had been made earlier by means of a panoramic radiograph.

According to a study conducted by Ericson and Kurol, once the permanent canine has been diagnosed in the ectopic position, the prevalence of root resorption of the adjacent permanent teeth, which commonly occurs silently and asymptomatically, is considerable. The case reported in this study exemplifies the consequences caused by the ectopic eruption of a permanent canine, which could have been avoided if the ectopic position of the canine had been diagnosed earlier and treated with the early extraction protocol of the deciduous canine and first molar, as suggested by Bonetti et al. [33], and if necessary, a later traction of the permanent canine.

Baccetti et al. evaluated the efficacy of two interceptive approaches for palatally displaced canines, extraction of primary canines alone or in association with the use of a cervical traction helmet and concluded that the additional use of a helmet resulted in successful eruption in 87.5% of the cases. The success rate with only canine extraction was 65.2%.

Early extraction of deciduous canines when there is a potential for impaction of the maxillary permanent canine seems to favor spontaneous eruption [3, 25, 34]. However, Parkin et al. concluded, based on a systematic review of the literature, that the extraction of deciduous canines between the ages of 10 and 13 years should be questioned due to the lack of evidence of their efficacy in favoring the eruption of the palatally ectopic maxillary permanent canine. Only two studies were included in the review, both of which had an elevated risk of bias.

For Becker and Chaushu the expansion of the upper arch and the verticalization of the roots of incisors or premolars are some of the effective measures in redirecting the canine, favoring its eruption. In addition, for Al Naqbi et al. [35], rapid maxillary expansion, trans palatal arch, and cervical-pull headgear are effective in intercepting canine impaction.

Akkuc et al. observed in their study, an incidence of maxillary permanent canine impaction of 3.43%, with resorption of maxillary lateral incisors in 33% of cases. Ectopic eruption of the permanent canine can also be related to the non-eruption of the permanent lateral incisor [36].

In addition to concerns regarding the consequences of ectopic eruption in the involvement of oral health, there are also psychological consequences, with an impact on the quality of patients' lives, as also concluded by Masood et al. This was noted in the patient in the present study because of the evident change in the smile line after the loss of the permanent central incisor. Untreated malocclusion had a significant negative impact on Oral Health-related Quality of Life [37].

Through this case report, it can be concluded that follow-up on the development and eruption of the permanent dentition are essential to detect possible changes early on and intervene when necessary, in an attempt to minimize, or even prevent, greater harm to the patient's health and well-being. The radiographic diagnosis was performed after the appearance of clinical signs, in this case characterized by the mobility of the central incisor and the rotation of the permanent lateral incisors, resulting in the exfoliation of the permanent central incisor, as well as the need for complex treatment, such as the re-anatomization of the canine, orthodontic and periodontal treatments, dental implants, and prostheses, as described in a similar case [38].

Although there is no protocol for radiographic examinations in dental pediatrics for follow-up on tooth development, this study should be reviewed by reference guidelines and manuals to evaluate the risks and benefits for patients.

## Data Availability

Data supporting this research article are available from the corresponding author or first author on reasonable request.
